# CD137 agonist induces gastric cancer cell apoptosis by enhancing the functions of CD8^+^ T cells via NF-κB signaling

**DOI:** 10.1186/s12935-020-01605-0

**Published:** 2020-10-20

**Authors:** Ben-Shun Hu, Tian Tang, Jun-Li Jia, Bi-Chen Xie, Tie-Long Wu, Ying-Yue Sheng, Yu-Zheng Xue, Hua-Min Tang

**Affiliations:** 1 School of Basic Medical Sciences, Nanjing Medical University, 101 Longmian Avenue, Jiangning District, Nanjing, 211166 People’s Republic of China; 2 Department of Hepatobiliary Surgery, Affiliated Hospital of Jiangnan University, Wuxi, People’s Republic of China; 3 Department of Pathology, Affiliated Hospital of Jiangnan University, Wuxi, People’s Republic of China; 4 Department of Gastroenterology, Affiliated Hospital of Jiangnan University, 200 Huihe Rd, Binhu District, Wuxi, 214000 People’s Republic of China

**Keywords:** CD137, Gastric cancer, CD8^+^ T cells, Immune microenvironment, Immune checkpoint

## Abstract

**Background:**

CD137 is a target for tumor immunotherapy. However, the role of CD137 in gastric cancer (GC), especially in inducing GC cell apoptosis, has not been studied.

**Methods:**

Foxp3^+^ and CD8^+^ T cells in GCs were investigated using immunohistochemistry (IHC). CD137 expression in GCs was detected using flow cytometry, IHC and immunofluorescence (IF). Peripheral blood mononuclear cells (PBMCs) and CD8^+^ T cells isolated from peripheral blood were stimulated with a CD137 agonist in vitro. CD8^+^ T cell proliferation and p65 expression was examined using flow cytometry. P65 nuclear translocation was analyzed using IF. IL-10, TGF-β, IFN-γ, perforin and granzyme B were detected using real-time quantitative PCR (real-time PCR). PBMCs and primary GC cells were cocultured and stimulated with a CD137 agonist in vitro. Apoptosis of primary GC cells was detected using flow cytometry.

**Results:**

Our data demonstrated that GC tumors showed characteristics of an immunosuppressive microenvironment. CD137 was predominantly expressed in CD8^+^ T cells in GCs and had a positive correlation with tumor cell differentiation. The CD137 agonist promoted CD8^+^ T cell proliferation and increased the secretion of IFN-γ, perforin and granzyme B, which induced primary GC cell apoptosis. Mechanistically, this study found that the CD137 agonist induced NF-κB nuclear translocation in CD8^+^ T cells.

**Conclusion:**

Our results demonstrated that a CD137 agonist induced primary GC cell apoptosis by enhancing CD8^+^ T cells via activation of NF-κB signaling.

## Background

Gastric cancer (GC) is a common malignant tumor. Chemotherapy and molecular-targeted therapy achieved limited improvements in survival [1, 2]. Immunotherapy is a new method of tumor treatment in addition to surgery, chemotherapy and radiotherapy [3, 4]. Therapeutic vaccines activate the initial T lymphocyte reaction, and enhances the activity of T lymphocytes [5]. Adaptive immunotherapy achieved an antitumor effect via the reinfusion of tumor-specific effector lymphocytes expanded in vitro [6]. Immunosuppressive agents showed a certain effect in the treatment of GC, but immune checkpoint agonists are less studied in GC.

CD137, also known as 4-1BB, is a member of the tumor necrosis factor (TNF) receptor family, and it is encoded by the TNF receptor superfamily member 9 (TNFRSF9) gene [7, 8]. Mouse CD137 is located at 75.5 cm on mouse chromosome 4, and it exhibits approximately 60% homology with human CD137 [9]. CD137 is primarily expressed in activated CD8^+^ and CD4^+^ T cells and regulatory T cells (Tregs) [10, 11]. Accumulated animal experiments demonstrated that mice with a systemic deletion of CD137 genes showed disordered immune homeostasis and lost the ability to fight against tumor immune memory. However, the role of CD137 in GC was not investigated [12].

CD137 expression is upregulated on antigen-presenting cells (APCs) as a result of the activation of T cells initiated by B7-1, B7-2 and antigenic peptides, which promote the production and secretion of cytokines via the activation of NF-κB [13, 14]. It is well established that CD137 induces TNFR-related factors TRAF1 and TRAF2 to form a heterotrimer, which activates mitogen-activated protein kinase (MAPK), β-catenin and AKT signaling and augments NF-κB nuclear translocation [15–17]. Notably, the activation of NF-κB contributes to the survival of CD8^+^ T lymphocytes by increasing the expression of the antiapoptotic genes Bcl-xL and bfl-l [11, 18]. However, whether CD137-mediated activation of NF-κB in CD8^+^ T lymphocytes induces GC cell apoptosis by enhancing the function of CD8^+^ T cells is not clear.

The most effective approach for CD137 agonist therapy is stimulation of the proliferation of CD8^+^ T cells by increasing the expression of IFN-γ and several granzymes [19]. The CD137 costimulatory signal is activated by a CD137 agonist or CD137L transfection, which induces cell proliferation, cytokine expression and bactericidal activity and supports T cell effector function [20]. CD137 agonists also inhibit the differentiation of conventional effector cells into Tregs, negatively regulates the activity of Tregs, or maintains the expansion and inhibition of Tregs [21]. However, the role of CD137 in Tregs of GC patients has not been investigated.

The present study demonstrated that GC tumors showed the characteristics of an immunosuppressive microenvironment. A CD137 agonist induced primary GC cell apoptosis by enhancing CD8^+^ T cells via activation of NF-κB signaling and increased the secretion of IFN-γ, perforin and granzyme B, but had little effect on Tregs in GC.

## Materials and methods

### Patients and specimens

For phenotypic assays, 23 fresh paired gastric cancerous, tumor margin and tumor-free gastric tissues (greater than 1-cm distance from the tumor), routinely paraffin-embedded for immunohistochemistry (IHC) and immunofluorescence (IF), were collected from 23 patients with GC who underwent surgery at our hospital between May 2019 and July 2020. Tumor infiltrating lymphocytes (TILs) isolated from above 23 fresh gastric cancerous tissues for flow cytometry, were collected. The clinical characteristics of the patients for phenotypic assay are listed in Table 1.

**Table 1:** Characteristics of patients for phenotypic data

	GC(23)
Sex	
Female	14
Male	9
Age (years)**	58.2 ± 3.6
Tumor size (cm)	
< 5	15
≥ 5	8
Smoking	
Yes	17
No	6
Drinking alcohol	
Yes	12
No	11
Tumor location	
Proxima	7
Distal	16
Histopathology	
Highly differentiated adenocarcinoma of GC	6
Poorly differentiated adenocarcinoma of GC	17
Lymph node metastasis	
N0–1	19
N2–3	4

*GC* gastric cancer

**Mean ± SD

For functional assays, peripheral blood from 18 patients with GC was collected before surgery. Paired 18 fresh gastric cancerous tissues were collected during surgery. The clinical characteristics of the patients for functional assays are listed in Table 2.

**Table 2:** Characteristics of patients for functional data

	GC(18)
Sex	
Female	7
Male	11
Age (years)**	65.2 ± 3.1
Tumor size (cm)	
< 5	6
≥ 5	12
Smoking	
Yes	10
No	8
Drinking alcohol	
Yes	9
No	9
Tumor location	
Proxima	4
Distal	14
Histopathology	
Highly differentiated adenocarcinoma of GC	5
Poorly differentiated adenocarcinoma of GC	13
Lymph node metastasis	
N0–1	12
N2–3	6

*GC* gastric cancer

**Mean ± SD

None of the patients who provided samples received preoperative radiotherapy or chemotherapy and were confirmed to have GC on postoperative pathology. The present study was performed in accordance with ethical standards and according to the declaration of the national and international guidelines. All the assays performed involving human peripheral blood and tissue samples (fresh gastric cancerous, tumor margin, and tumor-free gastric tissues) were approved by the Ethics Committee of Jiangnan University (No. LS2018021). All participants were aware of the study and signed an informed consent for publication.

### Antibodies and reagents

RNAlater^®^ was purchased from Ambion, USA. TRIzol was purchased from Invitrogen, USA. DEPC was purchased from Bio Basic Inc, Canada. The SYBR^®^ PrimeScript^®^ RT-PCR Kit was purchased from TaKaRa, Japan for two-step RT-PCR. PCR primers were designed by TaKaRa, Japan and synthesized by Yingjun Biotechnology Co., Ltd, China. An anti-CD137 rabbit mAb (#34549) used for IHC and IF and was purchased Cell Signaling Technology (CST, USA). An IHC detection reagent (HRP, rabbit, #8114) was purchased from CST, USA. An agonistic anti-CD137 mAb (#79097) was purchased from BPS Bioscience, USA. An anti-Foxp3 rabbit mAb (#12653) used for IHC was purchased from CST, USA. Anti-CD8 mouse antibody (#66868-1-Ig) for IHC and IF was purchased from the Proteintech group, China. MojoSort™ Magnet, MojoSort™ Human CD8 Nanobeads and MojoSort™ Human CD8 Cell Isolation Kit were purchased from BioLegend, USA. An NF-κB p65 rabbit mAb (#8242) for flow cytometry and IF was purchased from CST, USA. An anti-cytokeratin mouse mAb (#ab756) used for IHC was purchased from Abcam, England. A purified anti-human CD3 mAb (OKT3, #317326) for cell incubation and anti-CD45-PerCP (#368506), anti-CD3-FITC (#300406), anti-CD8-APC (#301014) and anti-CD137-APC (#309809) antibodies for flow cytometry were purchased from BioLegend, USA.

### IHC assay

Fresh tissues for phenotypic assays or collected primary GC cells for functional assays to test separation purity were fixed, dehydrated and paraffin embedded. Paraffin sections were dewaxed and rehydrated using a routine protocol [22]. The cells underwent antigen repair, neutralization of endogenous catalases, serum blocking, incubation with anti-CD137 rabbit mAb antibody (1:100, CST, USA), anti-Foxp3 rabbit mAb antibody (1:100, CST, USA), anti-cytokeratin mouse mAb antibody (1:100, Abcam, USA) and anti-CD8 mouse antibody (1:100, Proteintech group, China) at 4 °C overnight. Cells were incubated with a secondary antibody, and DAB was used for color development. Cells were counterstained, neutral gum sealed and observed according to a standard immunohistochemical operation procedure. PBS was used as a negative control. The stained sections were scanned using Panoramic MIDI. Image J was used to count positively stained cells. Two senior pathologists independently confirmed the results.

### IF assay

Paraffin sections of a specimen for phenotypic assays were dewaxed and sealed with 3% H_2_O_2_ for 10 min and heat-retrieved with 0.01 mmol/l citrate buffer (pH = 6.0) for 10 min at 95 °C. After natural cooling, the sections were blocked with goat serum (Beyotime Biotechnology, China) for 30 min and incubated with an anti-CD137 rabbit mAb (1:100, CST, USA) and anti-CD8 mouse mAb (1:100, Proteintech group, China) overnight in a water tank at 4 °C. After 1 h of rewarming, antigens were detected with an anti-rabbit IgG (H+L), F(ab’)2 Fragment (Alexa Fluor^®^ 594 Conjugate) and anti-mouse IgG (H+L), F(ab’)2 Fragment (Alexa Fluor^®^ 488 Conjugate) (both 1:500, CST, USA). The sections were incubated at 37 °C for 1 h, and DAPI was added. The sections were incubated in the dark for 5 min, sealed with 50% glycerol, and observed under a confocal microscope.

After slide preparation, cells for IF in NF-κB p65 nuclear translocation assay were fixed in 4% paraformaldehyde and penetrated using 0.5% Triton X-100 at room temperature for 20 min. The slides were blocked with goat serum (Beyotime Biotechnology, China) for 30 min and incubated with an anti-NF-κB p65 rabbit mAb (1:100, CST, USA) overnight in a water tank at 4 °C. After 1 h of rewarming, the primary antibodies were detected using an anti-rabbit IgG (H+L), F(ab’)2 Fragment (Alexa Fluor^®^ 594 Conjugate) (1:500, CST, USA) for 1 h. DAPI was added, and the sections were incubated in the dark for 5 min. Stained sections were observed under a fluorescence microscope.

### Isolation of TILs

Gastric cancerous tissues were cut into 1-mm-diameter pieces using ophthalmic surgical scissors, and the appropriate amount of tissue digestive solution containing 2 mg/ml type IV collagenase and 0.25 mg/ml hyaluronidase was added. The samples were transferred to a 15-ml centrifuge tube and digested in a shaker at 37 °C for 30 min. The cell suspension obtained from digestion was filtered with a 70-µm sieve, and the filtered liquid was collected in a 50-ml centrifuge tube. Ten milliliters of 40% Percoll was added, then 10 ml of 80% Percoll was added below the 40% Percoll. The tubes were centrifuged at 716*g* for 20 min. TILs were isolated between the 40% Percoll and 80% Percoll.

### Isolation of PBMCs and CD8^+^ T cells

After transferring 20 ml of blood from patients with GC into 50-ml centrifuge tubes, 10 ml of PBS was added to dilute the blood. The solution was mixed gently, and 10 ml of Ficoll lymphocyte separation solution was added to the bottom of 50-ml centrifuge tubes. Samples were centrifuged at 716*g* for 20 min, and lymphocytes were collected and washed twice with PBS for 5 min each time. Isolated PBMCs were washed with MojoSort™ buffer once. The experimental procedure for CD8 isolation protocol was performed according to the MojoSort™ Human CD8 T Cell Isolation Kit provided by BioLegend, USA.

### Isolation of primary GC cells

Fresh gastric cancerous tissues for functional assays were immersed in sterilized PBS containing 200 U/ml penicillin and streptomycin for 10 min, then washed with sterilized PBS containing 1000 U/ml penicillin and streptomycin 5 times. The specimens were immersed in sterilized PBS containing 200 U/ml penicillin and streptomycin for 10 min to remove blood and bacteria on the surface of the specimens. The tissue specimens were cut into 1-mm-diameter pieces using ophthalmic surgical scissors, and the appropriate amount of tissue digestive solution containing 2 mg/ml type IV collagenase and 0.25 mg/ml hyaluronidase was added. The samples were transferred to a 15-ml centrifuge tube and digested in a shaker at 37 °C for 30 min. The cell suspension obtained from digestion was filtered with a 70-µm sieve, and the filtered liquid was collected in a 50-ml centrifuge tube. Ten milliliters of Ficoll lymphocyte separation solution was added to the bottom of 50-ml centrifuge tubes, and the tubes were centrifuged at 716*g* for 20 min for lymphocyte removal. Cells at the bottom of 50-ml centrifuge tubes were collected, and erythrocyte lysate was added for 10 min to remove red cells. The cells were washed with sterile PBS containing 1000 U/ml penicillin and streptomycin 5 times.

### Primary GC cells and CD8^+^ T cells stained with CFSE

Primary GC cells and CD8^+^ T cells were collected and washed with PBS 3 times for 5 min each wash. Primary GC cells were treated with 1 ml of 5 µM CFSE and cultured in a 37 °C CO_2_ incubator for 15 min. One ml of fetal bovine serum was added to stop the staining for 1 min, and the cells were washed twice with PBS.

### Cell culture

CFSE-labeled CD8^+^T cells, PBMCs or/and CFSE-labeled primary GC cells isolated from GC patients were added to 96-well plates coated with a purified anti-human CD3 antibody (BioLegend, USA) at 5 µg/ml overnight to upregulate CD137 expression and cultured in DMEM with 10% FBS (Hyclone, USA).

### Flow cytometry

For CD137 detection, the PBMCs or TILs of GC patients were placed in flow tubes, and 5 µL each of an anti-CD45-PerCP antibody (BioLegend, USA), anti-CD3-FITC antibody (BioLegend, USA) and anti-CD137-APC antibody (BioLegend, USA) was added. The cells were incubated in the dark for 10 min and washed with PBS once. PBS (200 µL) was added for flow cytometry detection.

For examination of CD8^+^ T cells proliferation, CD8^+^ T cells of GC patients were placed in flow tubes and washed once with PBS. PBS (200 µL) was added for flow cytometry detection.

For NF-κB detection, CD8^+^ T cells from GC patients were treated with 10 µg/ml anti-CD137 mAb (BPS Bioscience, USA) for 72 h and placed in flow tubes. After washing once with PBS, a Fixation/Permeabilization Solution (BD Cytofix/Cytoperm™) was added at room temperature for 30 min. After washing once with PBS, an NF-κB p65 rabbit mAb (1:1000, CST, USA) was added. The cells were incubated in the dark for 1 h and washed once with PBS. Five microliters of anti-rabbit IgG (H+L), F(ab’)2 Fragment (Alexa Fluor^®^ 594 Conjugate) (1:500, CST, USA) was added, and the cells were incubated in the dark for 30 min. After washing once with PBS, 200 µL of PBS was added for flow cytometry detection.

### Primary GC cell apoptosis detection using flow cytometry

PBMCs (1 × 10^5^) and primary GC cells (2 × 10^4^, CFSE stained) were mixed, placed in anti-human CD3 antibody-coated 96-well plates containing 200 µL of 10% FBS (Hyclone, USA) DMEM, and treated with 10 µg/ml anti-CD137 mAb (BPS Bioscience, USA). Apoptosis in the GC cells was detected using flow cytometry after 72 h.

### Real-time quantitative PCR (real-time PCR)

Total RNA was extracted by TRIzol reagent (Invitrogen, USA) according to the manufacturer’s instruction, and cDNA was generated using a TaKaRa PrimeScript RT Reagent Kit (TaKaRa, Japan) according to manufacturer’s instructions. Quantitative real-time PCR was performed on ABI step-one plus (Applied Biosystems, USA) using the TB Green Premix Ex Taq (TaKaRa, Japan). Data were normalized to the expression of β-actin. Primer pairs used in this study are presented in Table 3.

**Table 3:** Real-time PCR primers description

Gene	Primer sequence	
	Forward (5′-3′)	Reverse (5′-3′)
β-actin	TGGCACCCAGCACAATGAA	CTAAGTCATAGTCCGCCTAGAAGCA
IL-10	GACTTTAAGGGTTACCTGGGTTG	TCACATGCGCCTTGATGTCTG
TGF-β	ACTTGCACCACCTTGGACTTC	GGTCATCACCGTTGGCTCA
IFN-γ	TCGGTAACTGACTTGAATGTCCA	TCGCTTCCCTGTTTTAGCTGC
Perforin	CGCCTACCTCAGGCTTATCTC	CCTCGACAGTCAGGCAGTC
Granzyme B	TGGGGGACCCAGAGATTAAAA	TTTCGTCCATAGGAGACAATGC

### Statistical analysis

Statistical analyses were performed using SPSS 26.0 software. The figures were plotted by GraphPad Prism 6 software. Continuous variables are shown as means ± standard deviations (SD). Categorical variables were shown as counts and percentages. Descriptive statistics were shown as mean (standard deviations, SD) or median (interquartile range) according to data distribution. Statistical analyses between different groups were performed by one-way ANOVA, with S–N–K for post hoc multiple comparisons. An unpaired two-tailed Student’s t test was used for comparisons between two groups. Significant p-values are labeled with one or more ‘*’, denoting *p < 0.05, **p < 0 .01, and ***p < 0 .001. A threshold of P < 0.05 was defined as statistically significant.

## Results

### Poor infiltration of CD8^+^ T cells but accumulation of Tregs in GCs

**Fig. 1:**
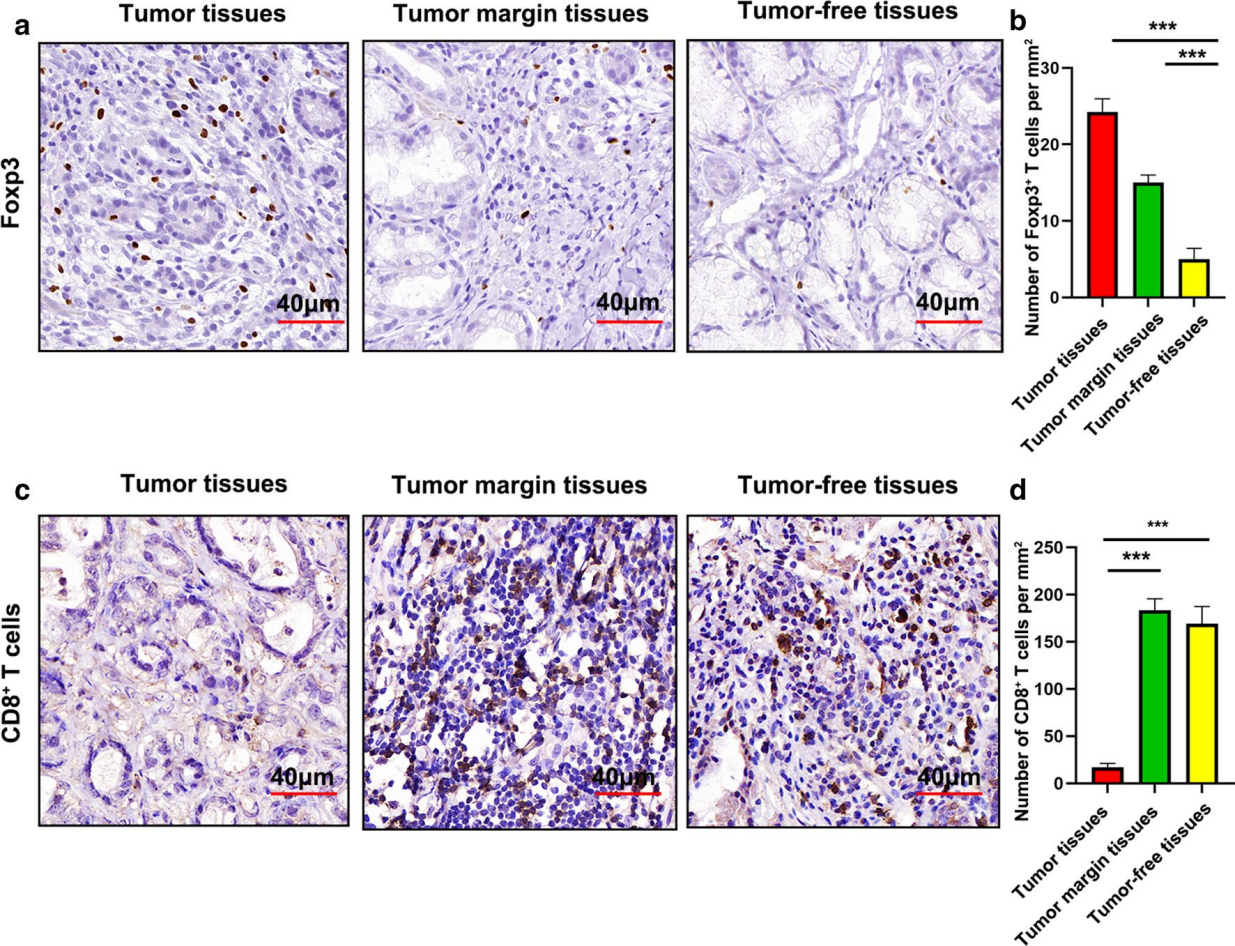
Poor infiltration of CD8^+^ T cells, but accumulation of Tregs, in GC. **a** Foxp3 expression the GCs was detected using IHC. **b** The cell densities of Foxp3 are depicted per square mm. **c** CD8 expression of GCs was detected using IHC. **d** The cell densities of CD8 are depicted per square mm. Bars = mean ± SD. ***P < 0.001

It is well known that CD8^+^ T cells in gastric cancer tissues are favorable for prognosis of GC patients and Foxp3^+^ Tregs are negatively associated with survival of GC patients. To compare the composition of CD8^+^ and Treg cells in the tumor tissues, tumor-free tissues and tumor margin tissues of GC patients, we analyzed the proportions of CD8^+^ T cells and Foxp3^+^ Tregs using IHC. Foxp3^+^ Tregs accumulated in the tumor (Fig. 1a and b), but most CD8^+^ T cells sequestered at the tumor margin and in tumor-free tissues (Fig. 1c and d). These data suggested that CD8^+^ T cells were excluded from the tumors, and Foxp3^+^ Tregs infiltrated into the tumors of GC patients.

### CD137 was highly expressed in differentiated tumor and primarily expressed in CD8^+^ T cells in GCs

**Fig. 2:**
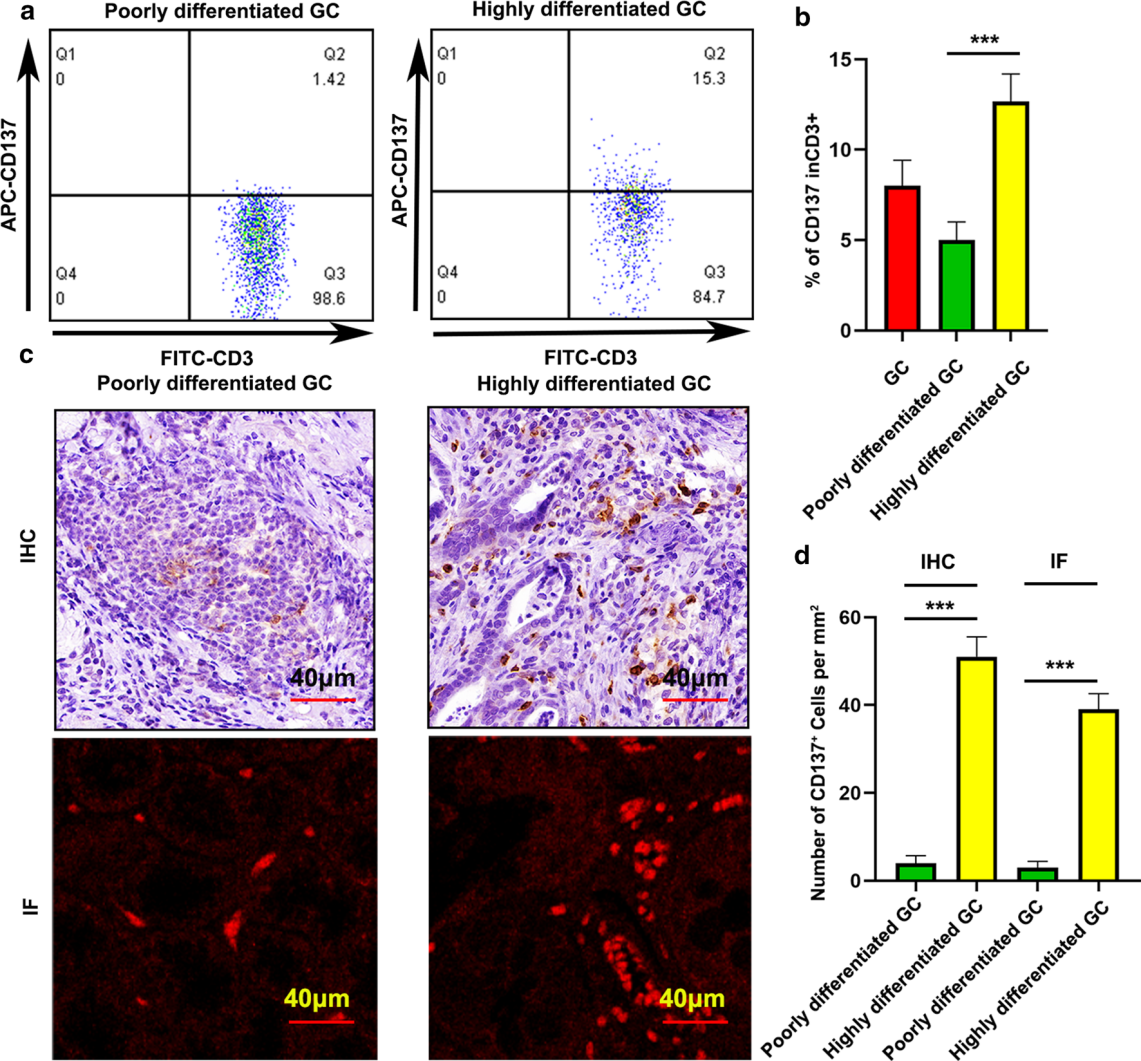
CD137 expression in TILs of GCs. **a** CD137 expression in GCs was examined using flow cytometry. **b** Statistical analysis of CD137 expression in different differentiations of GCs. **c** CD137 expression in GCs was measured using IHC and IF. **d** Statistical analysis of CD137 expression in different differentiations of GCs. Bars = mean ± SD. ***P < 0.001

**Fig. 3:**
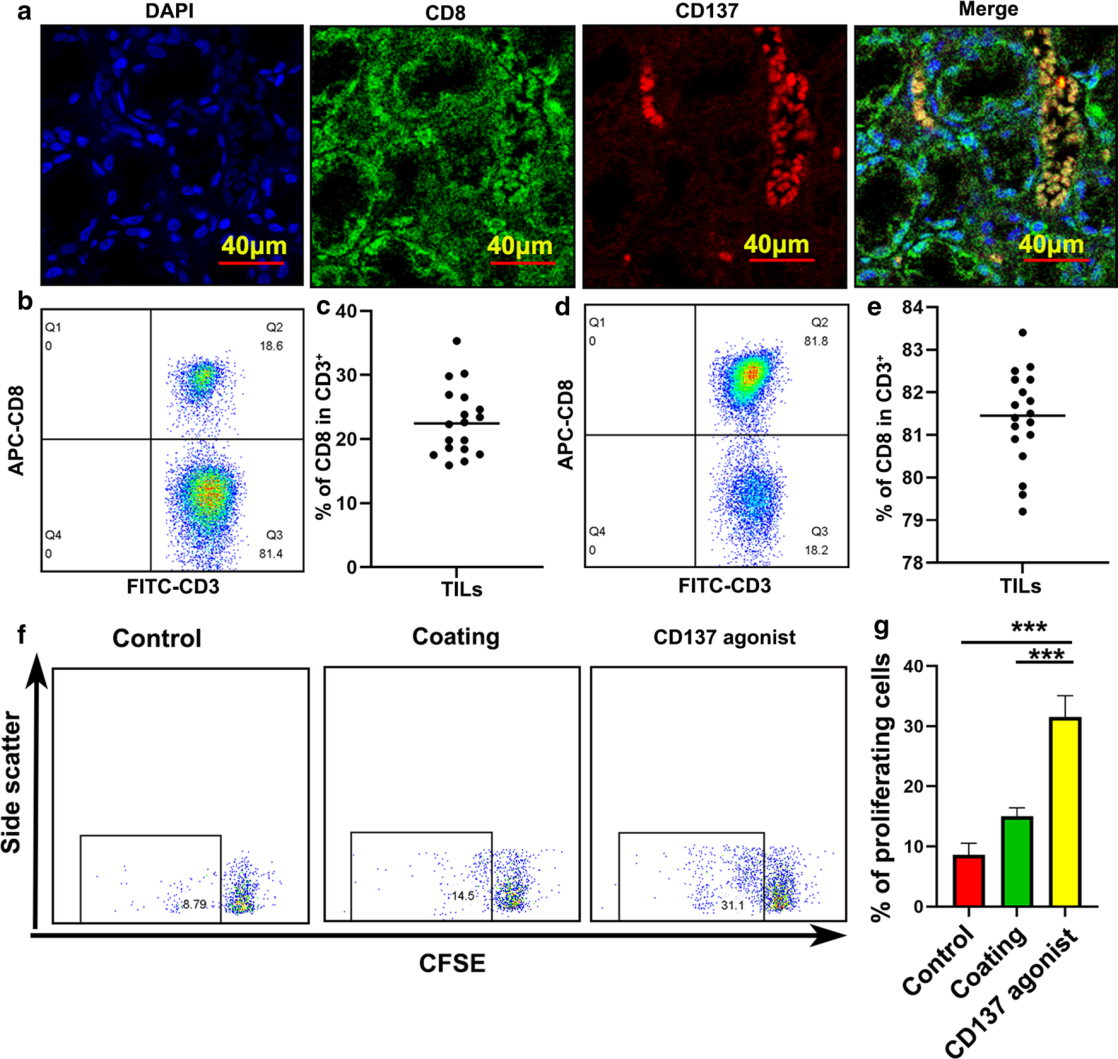
CD137 was primarily expressed on CD8^+^ T cells and a CD137 agonist enhanced CD8^+^ T cell proliferation. **a** CD137 expression was examined using IF. **b** The ratio of CD8 in CD3 before isolation. **c** The ratio of CD8 in CD3 of an individual patient before isolation is presented. **d** The ratio of CD8 in CD3 after isolation. **e**, Ratio of CD8 in CD3 of an individual patient after isolation is presented. **f** and **g** CD8^+^ T cell proliferation in response to a CD137 agonist. Bars = mean ± SD. ***P < 0.001

Previous studies demonstrated that CD137 is mainly expressed in activated CD8^+^ and CD4^+^ T cells and Tregs [10, 11]. However, CD137 expression in GCs has not been investigated. TILs are important components in tumor immune microenvironment. To analyze CD137 expression in GCs, we first examined CD137 expression in TILs of GC patients. Notably, CD137 was highly expressed in differentiated tumor (Fig. 2a and b). This result was confirmed using IHC and IF (Fig. 2c and d). IF showed that CD137 was primarily expressed in CD8^+^ TILs (Fig. 3a). Therefore, we focused on the function of CD137 on CD8^+^ T cells.

### An agonistic anti-CD137 mAb enhanced CD8^+^ T cell proliferation and increased the secretion of IFN-γ, perforin and granzyme B, but had little effect on Tregs in GC

**Fig. 4:**
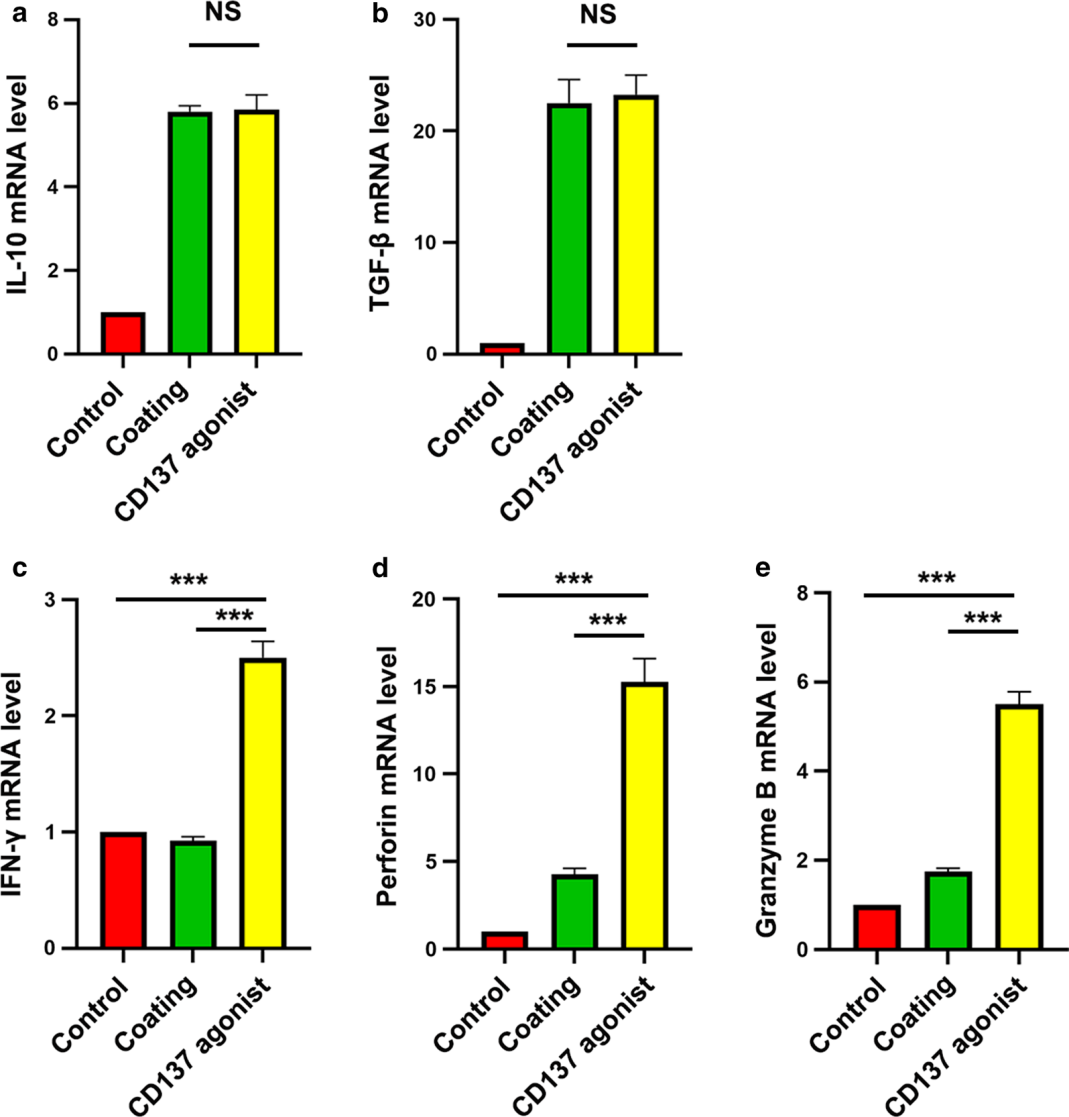
Functions of a CD137 agonist in the PBMCs of GC patients. **a** IL-10 detected using real-time PCR. **b** TGF-β detected using real-time PCR. **c** IFN-γ detected using real-time PCR. **d** Perforin detected using real-time PCR. **e** Granzyme B detected using real-time PCR. *NS* no significance. Bars = mean ± SD. ***P < 0.001

To determine the role of CD137 in the immune microenvironment of GC, CD8^+^ T cells were isolated from peripheral blood of GC patients (Fig. 3b–e) and stimulated with a CD137 agonist. The proliferation of CFSE-labeled CD8^+^ T cells was observed in the presence of an agonistic anti-CD137 mAb (Fig. 3f and g). We examined the overall effect of the CD137 agonist in GC patients. PBMCs were isolated from peripheral blood of GC patients. For Tregs, the secretion of IL-10 and TGF-β plays a role in maintaining immune tolerance. IL-10 and TGF-β were detected using real-time PCR. Our results showed that IL-10 and TGF-β levels were almost unchanged in the presence of a CD137 agonist (Fig. 4a and b). Notably, the CD137 agonist increased the production of IFN-γ, perforin and granzyme B (Fig. 4c–e) and secretion from CD8^+^ T cells in the PBMCs from GC patients. Taken together, these results demonstrated that the function of CD8^+^ T cells was enhanced in the presence of a CD137 agonist.

### NF-κB expression and nuclear translocation increased in CD8^+^ T cells after CD137 agonist treatment

**Fig. 5:**
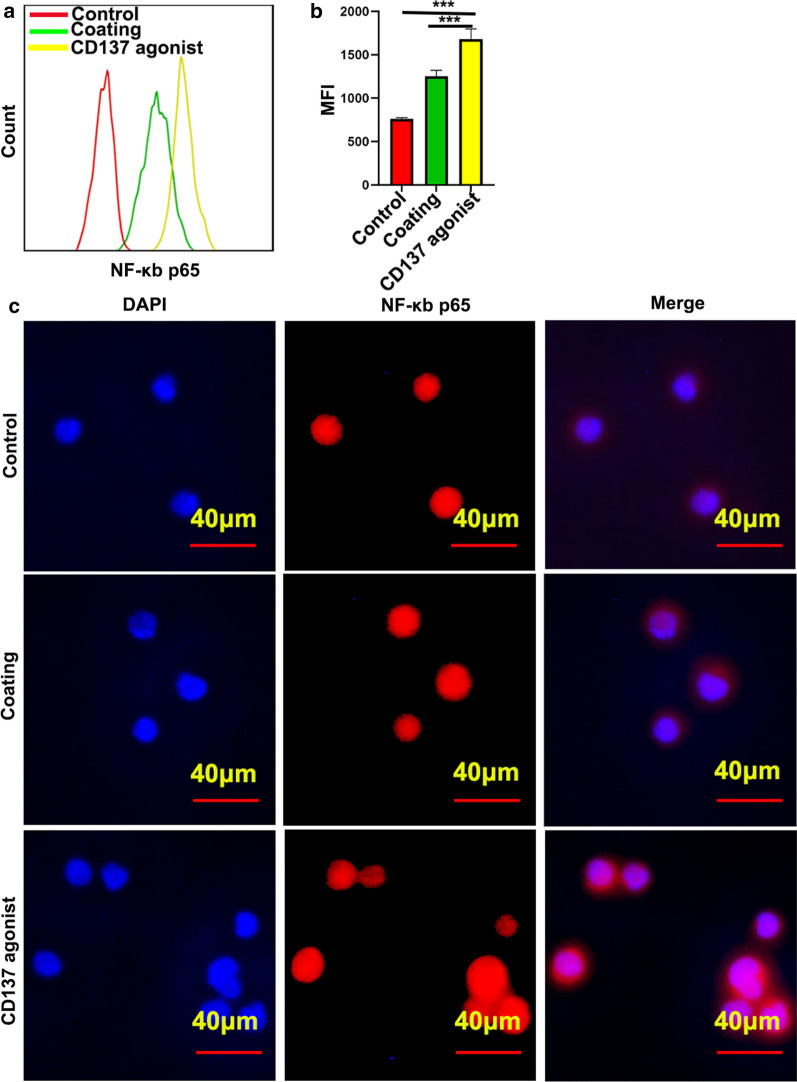
P65 expression and nuclear translocation were increased by the targeting of CD137. **a**, **b** The effect of a CD137 agonist on p65 expression was measured using flow cytometry. Bars = mean ± SD. ***P < 0.001. **c** The influence of a CD137 agonist on p65 translocation was detected using IF

The NF-κB transcription factor is important in regulating cell division and proliferation. The activation of NF-κB signaling is mainly induced by nuclear translocation of its subunits p65. After activation of NF-κB signaling, the levels and stability of the subunit p65 play an important role in the NF-κB signaling response. To examine the mechanism of CD137 in CD8^+^ T cells, NF-κB subunit p65 expression and nuclear translocation were detected. Flow cytometry showed that p65 expression increased significantly in CD8^+^ T cells in the presence of a CD137 agonist (Fig. 5a and b). Furthermore, IF showed that the CD137 agonist also induced p65 nuclear translocation in CD8^+^ T cells (Fig. 5c).

### A CD137 agonist induced apoptosis in primary GC cells

**Fig. 6:**
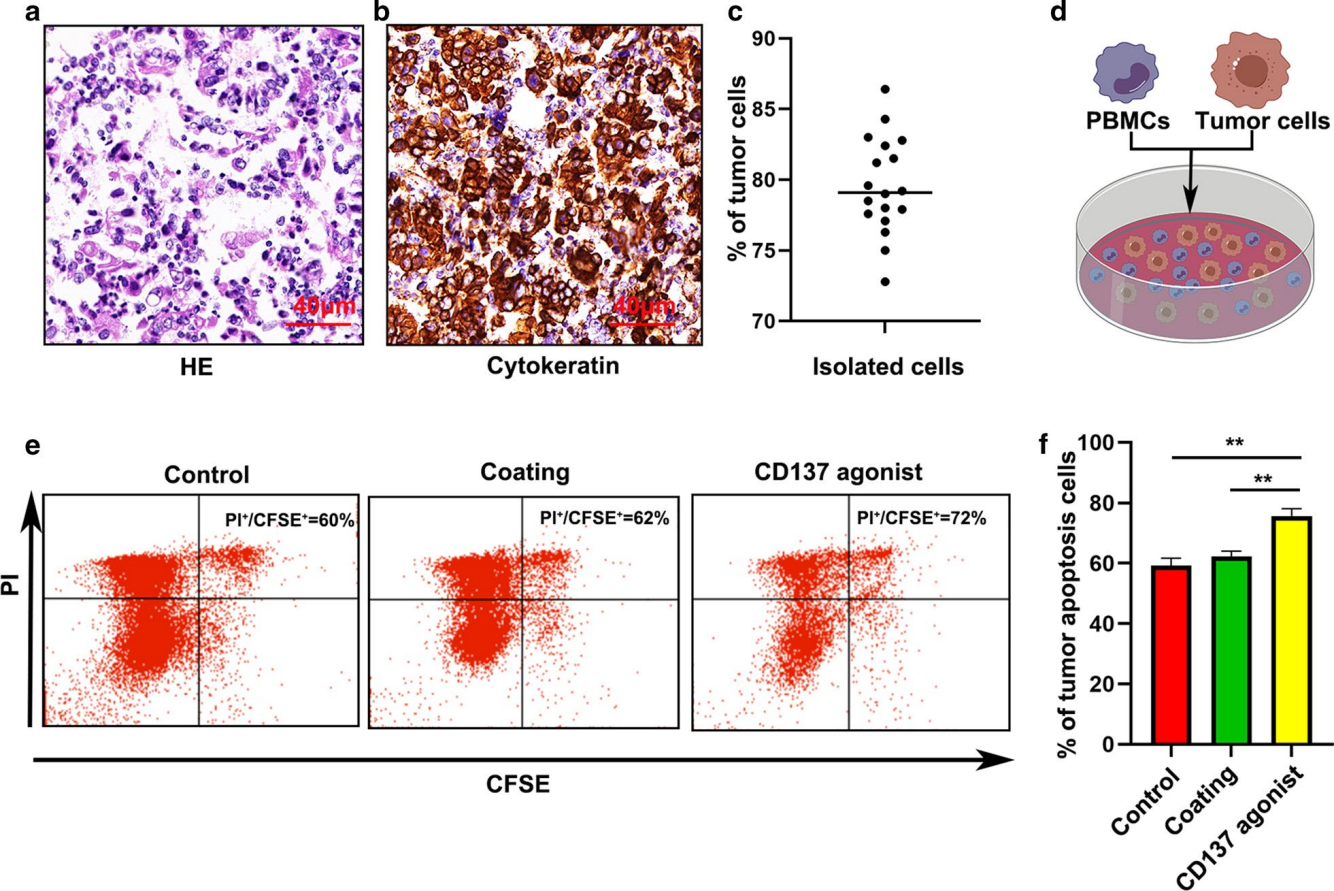
A CD137 agonist significantly induced apoptosis in primary GC cells. **a** The purity of isolated tumor cells was detected using HE staining. **b** IHC confirmed the purity of isolated tumor cells. **c** The purity of an individual patient after tumor isolation is presented. Values are means with standard error of the mean. **d** Apoptosis of primary GC cells was detected using flow cytometry. **e**, **f** Quantification of primary GC cell apoptosis. Bars = mean ± SD. **P < 0.01 and ***P < 0.001

We investigated the effect of the CD137 agonist on primary GC cells. HE staining combined with IHC (cytokeratin antibody to confirm tumors)was used to examine the purity of primary GC cells after isolation (Fig. 6a–c). To further study the function of the CD137 agonist in the immune microenvironment of GC, we cocultured PBMCs and CFSE-labeled primary GC cells at a ratio of 5:1 in vitro in the presence of 10 µg/ml of the agonistic anti-CD137 mAb. Flow cytometry was used to detect primary GC cell apoptosis after 72 h. Compared to control treatment, the CD137 agonist induced apoptosis in the primary GC cells (Fig. 6d and e).

## Discussion

The morbidity and mortality of GC are high in the world, and the clinical therapeutic effect of monoclonal antibodies against a single target in GC is limited [6, 23, 24]. To improve the curative effect and reduce drug resistance, the identification of specific monoclonal antibodies for the treatment for GC is urgent [25, 26]. Immune escape is an important process of tumor development. We also found that the immunosuppressive microenvironment excluded CD8^+^ T cells from the tumors, but Foxp3^+^ Tregs infiltrated into the tumors of GC patients. CD137/CD137L targeted therapy is effective against melanoma, leukemia and other tumors [27]. The present study examined the effects of CD137 on the immune microenvironment of GC to provide new ideas for treatment.

We demonstrated that CD137/CD137L activation signaling promoted the activation and proliferation of tumor-specific T cells, increased the secretion of cytokines, and protected T cells from activation-induced cell death [28, 29]. The intraperitoneal administration of an anti-CD137 monoclonal antibody eliminated tumors that were established via subcutaneous inoculation of Agl04A sarcoma or 10815 mast cells in mice on the third and seventh day after inoculation, respectively [25]. The enhanced immune response was primarily mediated by CD8^+^ T cells that were activated by the anti-CD137 monoclonal antibody and accompanied by a significant enhancement in tumor-specific cytotoxic T lymphocyte (CTLs) activity [30]. For CD4^+^ T cells, CD137/CD137L signal transduction induces cell expansion but does not prolong cell survival [31]. CD137 is primarily expressed on the surface of activated T cells, and our study found that CD137 was predominantly expressed on the surface of CD8^+^ T cells in the GC immune microenvironment and may positively correlate with tumor differentiation.

CD137 has a more restricted number of TRAF family members involved in its regulation, and only TRAF1, TRAF2, and TRAF3 interact with and control CD137 activity [32]. Recruitment of the CD137 signalosome by K63-polyubiquitinated TRAF2 is a kinase complex composed of transforming growth factor beta-activated kinase (TAK)-1, which phosphorylates the inhibitor of nuclear factor κ-B kinase (IKK)-β and leads to the activation of canonical NF-κB [33]. Our results showed that a CD137 agonist enhanced CD8^+^ T cell proliferation. A CD137 agonist also increased p65 expression and induced p65 nuclear translocation in CD8^+^ T cells, which suggests a mechanism for enhancing the functions of CD8^+^ T cells to induce gastric cancer cell apoptosis by a CD137 agonist.

**Fig. 7:**
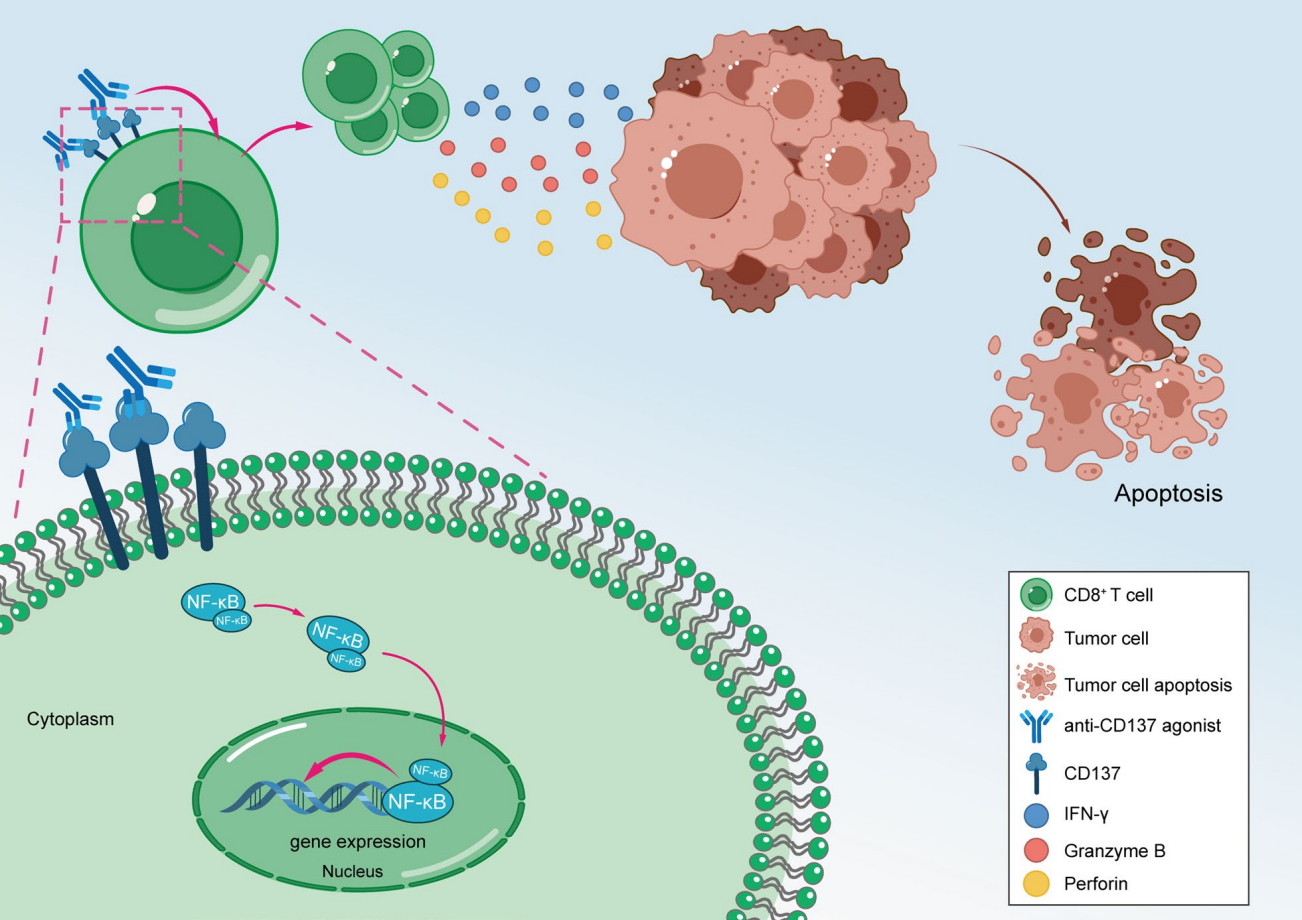
Schematic diagram of a CD137 agonist in inducing GC apoptosis via enhancing CD8^+^ T cell functions

Tregs plays a critical role in maintaining immune tolerance through IL-10 and TGF-β secretion [34]. CD8^+^ T cells are mounting to cancer responses by releasing perforin, granzyme B, and IFN-γ [35]. We examined the effects of a CD137 agonist on Tregs, and the results suggested that IL-10 and TGF-β levels were almost unchanged in the presence of a CD137 agonist. Interestingly, our study demonstrated that the CD137 agonist increased the secretion of IFN-γ, perforin and granzyme B in the CD8^+^ T cells. Furthermore, The CD137 agonist significantly induced apoptosis in cocultured primary PBMCs and primary GC cells, which suggests that a CD137 agonist is an adaptor in the immune microenvironment of GC (Fig. 7).

## Conclusions

CD8^+^ T cells were excluded from tumors, and Foxp3^+^ Tregs infiltrated into the tumors in GC patients. CD137 was primarily expressed on CD8-positive T cells in the GC immune microenvironment. A CD137 agonist enhanced CD8^+^ T cells proliferation via NF-κB signaling and increased the secretion of IFN-γ, perforin and granzyme B but had little effect on Tregs in GC. A CD137 agonist induced apoptosis in primary GC cells. Our study provide the theoretical basis for the treatment of GC employing CD137 agonists.

## Data Availability

Data and materials are available for sharing if needed.
